# Characteristics of patients with recurrent retinoblastoma: a survival analysis

**DOI:** 10.1186/s12885-024-12058-3

**Published:** 2024-03-04

**Authors:** Nan Li, Yi-Zhuo Wang, Yi Zhang, Wei-Ling Zhang, Dong-Sheng Huang

**Affiliations:** Department of Pediatrics, Beijing Tongren Hospital, Capital Medical University, Yizhuang Economic and Technological Development Zone, No. 2, Xihuan South Road, Beijing, 100176 China

**Keywords:** Retinoblastoma, Recurrence, Survival analysis

## Abstract

**Background:**

Management guidelines and corresponding survival data for patients with recurrent retinoblastoma (RB) are lacking. This study aimed to summarize the clinical characteristics of patients with recurrent RB and analyze their survival outcomes.

**Methods:**

We retrospectively analyzed 68 patients with recurrent RB who underwent treatment in our institution from January 2016 to December 2020. Patients were grouped according to location of recurrence: intraocular, orbital, and distant metastasis.

**Results:**

The male:female ratio was 1.3:1 and the median age at recurrence was 37.5 months (range, 30.3–62.8). The number of patients in the intraocular recurrence, orbital recurrence, and metastasis groups was 13 (19.1%), 23 (33.8%), and 32 (47.1%), respectively. Thirty patients died, 36 were alive at last follow-up, and two were lost to follow-up. Eye enucleation was performed in 94.1% of patients. Five-year overall survival in patients with intraocular recurrence, orbital recurrence, and metastasis was 84.6%, 69.6%, and 31.3%, respectively (*P* = 0.001). Most deaths occurred within 2 years of recurrence. Presence of high-risk pathological factors, central nervous system invasion, and absence of combination therapy were independent predictors of worse 5-year overall survival.

**Conclusion:**

The rate of eye preservation in survivors of recurrent RB was very low. Although 5-year overall survival in patients who underwent treatment for intraocular and orbital recurrence was high, it was low in those with metastasis. RB patients may need lifelong follow-up for recurrence and secondary malignancy.

## Introduction

Retinoblastoma (RB) is the most common primary eye cancer in children and the second most common intraocular cancer worldwide. It accounts for 4% of all malignant tumors in children [1]. China reports approximately 1000 new cases of RB every year [2]. Favorable RB outcomes depend on early diagnosis and timely treatment [3]. Survival in RB patients has been improving in conjunction with the development of new treatments. Recurrence monitoring and management are particularly important in RB patient follow-up. Extraocular dissemination and metastasis are also important factors affecting RB outcomes.

Reese published the first study on RB recurrence in 1948 and estimated an orbital recurrence rate of 72% [4]. A recent 2019 review of recurrent RB noted that recurrence is usually intraocular and that reported recurrence rates range from 6 to 45% [5]. Although most recurrences occur within 3 years of treatment, [6] late recurrence after more than 10 years has been reported [7]. Currently, management guidelines and corresponding survival data for patients with recurrent RB are lacking. This study aimed to summarize patient and tumor characteristics in children with recurrent RB treated in our institution. We also analyzed the survival outcomes of patients with recurrent RB and the factors affecting their survival rate, which may be valuable for the follow-up and long-term management of RB patients.

## Materials and methods

Sixty-eight children with recurrent RB who were treated in the pediatric ward of Beijing Tongren Hospital from January 2016 to December 2020 were analyzed. Data regarding age, gender, clinical characteristics, tumor laterality, family history, tumor stage, treatment, and survival status were recorded. Intraocular RB was staged according to the International Intraocular Retinoblastoma Classification (stages A–E) [8]. Extraocular disease was assessed using the RB clinical staging and TNM staging systems [1]. Patients were grouped according to location of recurrence: intraocular (cT0-cT3N0M0), orbital (cT4N0M0), and distant metastasis (cN1, cM1, or pM1).

After recurrent disease was diagnosed using fundus imaging or magnetic resonance imaging, patients underwent bone marrow puncture, lumbar puncture, lymph node ultrasonography, head or local magnetic resonance imaging, positron emission tomography, and pathological examination of any metastatic foci for staging and treatment decision making purposes. Serum neuron-specific enolase (NSE) level was examined monthly (or before each chemotherapy cycle). Follow-up imaging of the eye and metastatic lesions was performed every 2 to 3 months.

Stage A and small stage B tumors underwent local treatment. Large stage B tumors and stage C and D tumors were treated using local treatment and intravenous chemotherapy (IVC). Enucleation of the eye was performed for stage E tumors and in patients with eye preservation failure. Enucleation was also combined with IVC in some cases. Children with extraocular RB were treated with surgery, IVC, and radiation therapy based on their condition. If the tumor extended intracranially, high-dose IVC with radiation therapy and intrathecal chemotherapy was administered [9]. Patients with systemic metastasis were treated using enhanced IVC combined with autologous stem cell transplantation (ASCT) [10]. Multidrug combinations were used for IVC including carboplatin, etoposide, vincristine, cisplatin, and cyclophosphamide. Dosages were adjusted according to the child’s chemotherapy tolerance and degree of bone marrow suppression. Individualized treatment was used for complex cases.

Patients were followed via outpatient clinic visits and phone calls through December 2022. Outcome was classified as complete remission, partial remission, progressive disease, recurrence, or death. Overall survival (OS) after recurrence was defined as the time from RB recurrence diagnosis to the time of death from any cause. Patients’ guardians provided written informed consent. The study was approved by Beijing Tongren Hospital's Medical Ethics Committee.

Statistical analyses were performed using SPSS software version 25.0 (IBM Corp., Armonk, NY, USA). Survival was analyzed using the Kaplan–Meier method and compared using the log-rank test. Variables analyzed included recurrence location, age of onset, monocular or binocular disease, interval between recurrences, serum NSE level, pathological high-risk factors, site of metastasis, enucleation, and use of a combined treatment regimen (combination of one or more of the following: surgery, radiation therapy, ASCT, and IVC). Factors found to be significant in the univariate analyses were used in a multivariate Cox proportional hazards regression model to calculate hazard ratios (HRs) with 95% confidence intervals. *P* < 0.05 was considered significant.

## Results

Among the 68 study patients, the disease was monocular in 49 and binocular in 19 (a total of 87 eyes were affected). Sex was male in 38 and female in 30 (1.3:1 male:female ratio). The main symptoms at onset were leukocoria in 54 patients, decreased vision in 12, and strabismus in 10. Median age at RB onset was 23.0 months (range, 15.0–33.5). Median age at RB recurrence was 37.5 months (range, 30.3–62.8). Six patients (8.8%) were over age 7 years at the time of recurrence; the oldest patient was 11 years old. At the time of the initial diagnosis, RB was intraocular in 61 patients, extraocular in six, and metastatic in one. Stage was E or D at onset in all patients. Treatment was delayed or non-standard in 23 patients (33.8%).

Characteristics of the study patients and 696 patients with non-recurrent RB hospitalized in the same period (excluding those with trilateral RB) are shown in Table 1. Although the male:female ratio was similar between the groups, median age and proportion of patients with binocular disease were higher in the patients with recurrent RB.

**Table 1:** Characteristics of patients with recurrent retinoblastoma and those with non-recurrent disease

	Number	Sex	Median age(mon)	Mon/Bin	Proportion (%)		
		M/F			Intraocular	Orbital	Distant metastasis
Non-recurrent^a^	696	1.3:1	19.0	2.6:1	90.7	7.5	1.9
Recurrent	68	1.3:1	23.0	2.4:1	89.7	8.8	1.5

*M* Male, *F* Female, *Mon* Monocular, *Bin* Binocular

^a^patients with trilateral RB were not included

Characteristics of the study patients grouped according to location of recurrence are shown in Table 2. Recurrent disease in the patients with intraocular recurrence was found mainly by follow-up fundus imaging or magnetic resonance imaging of the eye (11 of 13 patients, 84.6%) rather than by clinical manifestations. Children with metastatic recurrence frequently exhibited eye protrusion and redness (12 of 32 patients, 37.5%) or atypical symptoms such as unexplained fever, metastatic masses, and seizures. Among the 49 children with monocular disease, recurrence was intraocular in five, orbital in 18, and metastatic in 26. Among the 19 patients with binocular recurrence, recurrence was intraocular in eight, orbital in five, and metastatic in six.

**Table 2:** Patient characteristics and overall survival rates according to location of recurrence

Stages	Number (n)	Age (mon)	NSE level (ng/ml)	Recurrent manifestations(%)		OS(%)	
	(Mon/bin)			Fundus/MRI	Protrusion /redness	1-year	5-year
Intraocular	13(5/8)	15.0	19.8	84.6	15.4	100	84.6
Orbital	23(18/5)	20.0	25.8	43.4	39.1	91.3	69.6
Distant metastasis	32(26/6)	25.0	174.3	9.4	37.5	68.3	31.3
Total	68(49/19)	23.0	35.6	35.3	33.8	85.2	54.9

*Mon* Monocular, *Bin* Binocular, *NSE* Neuron-specific enolase, *MRI* Magnetic resonance imaging, *OS* Overall survival

Median NSE level in patients with recurrent RB was 35.6 ng/mL (range, 21.1–326.0); < 16.2 ng/mL is considered normal in our institution. NSE level significantly differed between patients with intraocular, orbital, and metastatic recurrence (19.8, 25.8, and 174.3 ng/mL, respectively; *H* = 27.441; *P* = 0.000). The most common site of metastasis was the orbit (35 patients, 51.5%), followed by the central nervous system (CNS; 24 patients, 35.3%), bone (25.0%), bone marrow (17.6%), and soft tissue (13.2%). Soft tissue metastases were mainly located in the superficial lymph nodes and parotid gland. Twenty-eight eyes were removed after recurrence was diagnosed owing to presence of high-risk pathological factors. The most common high-risk pathological factors were extensive choroid involvement (46.4%), optic nerve involvement (42.9%), and anterior segment involvement (42.9%).

Because multiple relapses occurred in some patients, only management and treatment after the first recurrence were analyzed. Among the 13 patients (19.1%) with intraocular recurrence, eight underwent enucleation combined with IVC and five underwent eye preservation treatment. One patient in each treatment subgroup died and one was lost to follow-up. Overall mortality for patients with intraocular recurrence was 15.4%.

Among the 23 patients with orbital recurrence (33.8%), all underwent IVC. Seventeen underwent combined surgical treatment and four underwent orbital radiation therapy (36–48 Gy). Six patients who experienced orbital recurrence after enucleation underwent orbital evisceration and IVC treatment; among these, complete remission was achieved in five. Ultimately, seven patients with orbital recurrence died (30.4% mortality).

Among the 32 patients with distant metastasis (47.1%), only 11 were alive at last follow-up (65.6% mortality). Thirteen were treated with IVC alone for various reasons (patient condition, family wishes); among these, 10 died and one was lost to follow-up. Another three patients were treated with ASCT and IVC; one of these patients died. Among the 16 who underwent IVC combined with radiation therapy and/or surgical treatment, 10 died.

Complete remission was achieved after treatment of recurrence in 43 patients overall (63.2%). A second recurrence developed in nine: seven died, one was lost to follow-up, and one survived. One patient developed a secondary tumor (B-cell acute lymphocytic leukemia). Mean follow-up after recurrence was 51.5 months (range, 41.3–81.0). Thirty patients died overall (44.1% mortality).

**Fig. 1:**
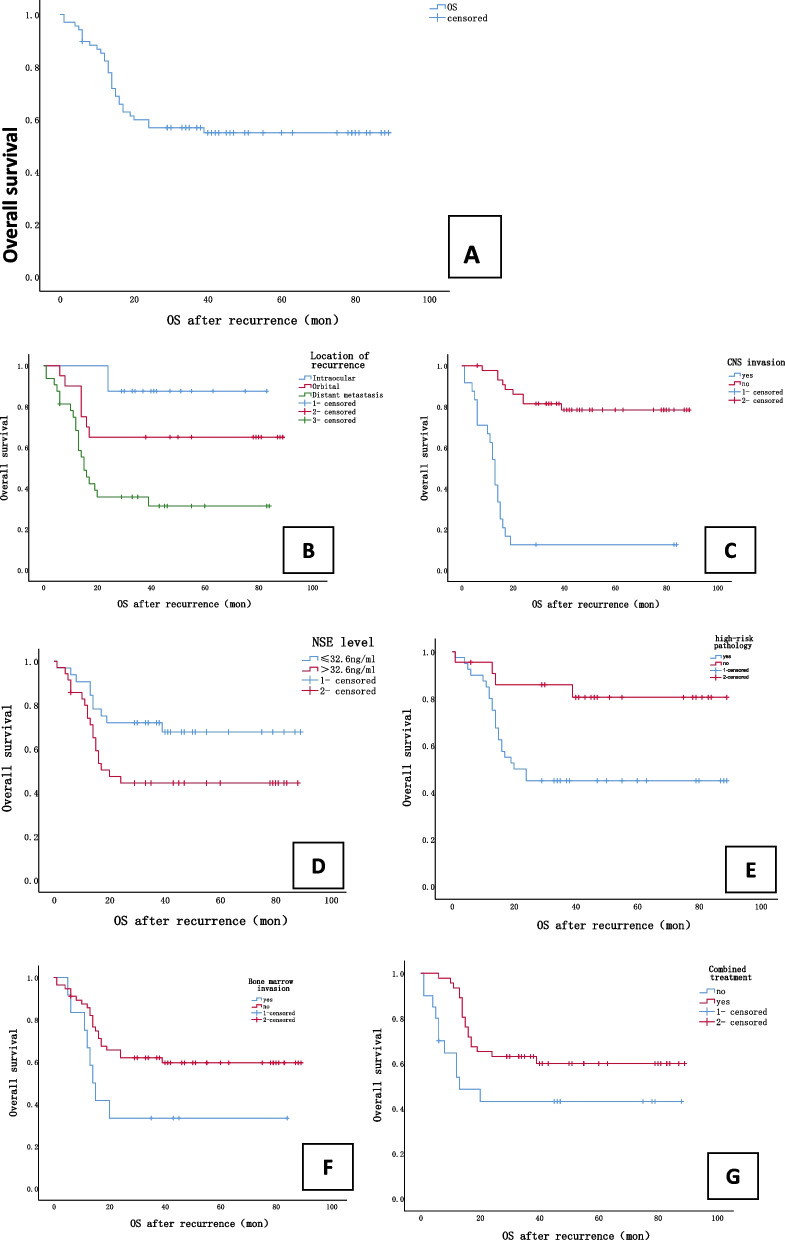
Kaplan–Meier curves of overall survival after recurrence in (**A**) all patients and according to (**B**) location of recurrence, **C** CNS invasion, **D** NSE level, **E** high-risk pathology, **F** bone marrow invasion and (**G**) combined treatment. OS, overall survival, Mon, month

Among the patients who died, 21 showed clinical evidence of CNS metastasis (70%). Median OS after recurrence was 33.0 months (range, 14.0–55.0). One-year and 5-year OS rates were 85.2% and 54.9%, respectively (Fig. 1-A). Five-year OS significantly differed between patients with intraocular, orbital, and metastatic recurrence (84.6%, 69.6%, and 31.3%, respectively; *P* = 0.001; Table 2; Fig. 1-B). Death mainly occurred within 2 years of recurrence (85.2%). Most patients (94.1%) had at least one diseased eye removed before or after recurrence. Among the 36 patients who ultimately survived (excluding two lost to follow-up), single-eye enucleation was performed in 32 (88.9%) and double-eye enucleation in two (5.6%).

In the univariate survival analyses, location of recurrence (*P* = 0.01; Fig. 1-B), presence of CNS invasion (*P* = 0.0001; Fig. 1-C), elevation of serum NSE level to twice the normal value (*P* = 0.044; Fig. 1-D), presence of high-risk pathological factors (*P* = 0.008; Fig. 1-E), presence of bone marrow invasion (*P* = 0.048; Fig. 1-F), and absence of combined treatment regimen (*P* = 0.41; Fig. 1-G) were significant predictors of 5-year OS. The multivariate Cox regression model showed that presence of high-risk pathological factors (HR, 0.243; *P* = 0.019), CNS invasion (HR, 0.059; *P* = 0.000), and the absence of combination therapy (HR, 0.349; *P* = 0.034) were independent predictors of worse 5-year OS (Table 3).

**Table 3:** Multivariate Cox regression analysis of overall survival

	***P*** **value**	**Exp(B)**	**95% confidence interval**	
			Lower limit	Upper limit
**Location of recurrence**	.422	1.459	.581	3.666
**Elevation of serum NSE level**	.444	1.456	.557	3.802
**Presence of high-risk pathological factors**	**.019**	.243	.074	.794
**Presence of CNS invasion**	**.0001**	.059	.017	.200
**Presence of bone marrow invasion**	.916	1.057	.375	2.980
**Absence of a combined treatment regimen**	**.034**	.349	.132	.923

*NSE* Neuron-specific enolase, *CNS* Central nervous system

## Discussion

As treatment methods have improved, survival and eye preservation rate in patients with RB have improved as well. Today, the prognosis of early-stage RB patients is good [11]. Metastasis and secondary malignancy are the main causes of death in RB patients and RB recurrence is a key issue. Outcomes when recurrence is limited to the eye are generally good; survival in these patients exceeds 90% [12]. However, reported mortality rates in RB patients with tumor infiltration of the orbit range from 25 to 100% [13]. Prognosis is also quite poor in RB patients with distant metastasis [14].

Previous studies have reported that patients with recurrent RB are more likely to be younger at diagnosis and have bilateral disease, family history of RB, and *RB1* gene mutations; other risk factors for recurrence include subretinal seeding, vitreous seeding, tumor basal diameter > 16 mm at diagnosis, macular location, and increased tumor thickness [5, 15]. In our study, sex distribution in RB patients with and without recurrence was similar. Although the median age was slightly higher in patients with recurrence, the difference was not significant. The proportion of patients with bilateral disease was somewhat higher in patients with recurrence, consistent with previous reports [5]. Diagnosis and treatment were delayed in nearly one-third of our patients, which is a common issue with RB management in developing countries that may explain the relatively poor outcomes.

Recurrent RB should be treated based on the degree of disease involvement; however, currently there is no unified management consensus. Local treatment can be considered for early recurrent intraocular disease, while for mid- to late-stage disease, combined treatment or surgical treatment is necessary [5]. The mortality rate of patients with intraocular recurrence treated with eye protection therapy in our study was higher than in previous reports. Therefore, eye protection therapy should be considered with caution in these patients.

Orbital metastasis is the most common extraocular site of involvement in RB patients. Simple orbital recurrence requires IVC combined with surgical treatment, radiation therapy, and other treatments. In our study, six patients with recurrence after eye extraction underwent orbital evisceration, one of whom patient died. The fact that the mortality rate was lower in these patients than in all patients with orbital recurrence suggests that secondary orbital evisceration has a role in treating patients with extensive orbital metastases and a limited response to IVC. Patients with extensive (> 5 mm) optic nerve infiltration or involvement of the optic nerve stump may have a poor outcome despite combined treatment [9]. Although ASCT treatment may improve outcome in these patients, [13] this has not yet been examined in patients from China.

Combined systemic IVC and radiation therapy has been successful in treating soft tissue, bone, and CNS metastases in several small-scale studies [9, 16–19]. We recommend a comprehensive management plan and personalized treatment for metastatic RB. In addition to IVC, combined surgical treatment and radiation therapy can provide local control for orbital disease and optic nerve involvement. For regional lymph node or parotid gland infiltration, local tumor resection (Supplemented with radiation therapy and chemotherapy) can establish the diagnosis of metastasis and reduce the tumor burden to improve outcomes. Because resection of bone and CNS metastasis can be difficult, local control at these sites is usually achieved using radiation therapy. Although ASCT may improve survival to 50% to 70% after high-dose chemotherapy in patients with metastatic and extraocular RB, patients with CNS metastasis still have a poor prognosis [10, 11, 20, 21]. Three patients in our study underwent chemotherapy combined with ASCT: two survived and one with CNS metastasis experienced recurrence and died. Unfortunately, the role of ASCT in patients with CNS metastasis may be limited [10]. Currently, targeted therapy, immunotherapy, and oncolytic viruses are non-chemotherapy treatment options for RB. Many new potential treatments are still in the experimental stage [22].

Although RB recurrence detrimentally affects patient survival, few studies have explored survival in this patient population. In our study, overall mortality was 44.1% and 5-year OS in patients with intraocular, orbital, and metastatic recurrence was 84.6%, 69.6%, and 31.3%, respectively. High-risk pathological factors, CNS invasion, and absence of combination therapy were independent predictors of worse OS after recurrence. If RB metastases in the CNS involve an intracranial segment of the optic nerve or the optic chiasm, a relatively good outcome is possible because local control can be achieved through surgery, intrathecal injection, or extracerebral radiation therapy [23]. However, outcomes in patients with cerebrospinal fluid dissemination and metastasis are extremely poor; cure is almost never achieved. Current treatments for RB patients with CNS metastasis are limited and their effectiveness is low. Early diagnosis, standardized management, and active treatment interventions for patients with high-risk pathological factors are essential in reducing metastasis and mortality rates.

For late and recurrent RB, enucleation of the eye plays a vital role in disease control and mortality reduction [24]. An international study reported single- and double-eye enucleation rates of 66% and 3%, respectively [24]. In our study, 94.1% of patients had at least one diseased eye removed. Single- and double-eye enucleation rates among RB survivors were 88.9% and 5.6%, respectively. This high rate of eye extraction in patients who survive RB recurrence causes disability and seriously affects quality of life because of visual, cosmetic, and psychological factors [25]. These individuals need a considerable degree of social support and intervention, which requires efforts at both the private and public levels of society.

Follow-up of RB is essential. Regular imaging of the fundus and eye are mandatory after treatment to detect early recurrence. Clinical symptoms such as eye redness, protrusion, or recurrent fever usually indicate advanced RB. Patients with recurrence require follow-up after treatment to monitor for recurrence and development of a second primary malignancy. Those who experience multiple recurrences may have a worse outcome; in our study, these patients had a 77.8% mortality.

This study had several limitations. Early-stage cases of RB recurrence were not included as most of these patients received local treatment and did not require pediatric or inpatient treatment (surgery or IVC); therefore, selection bias was almost certainly present and the reported survival rates of patients with intraocular recurrence were probably underestimated. Furthermore, our findings may not be generalizable to patients with non-recurrent RB.

## Conclusions

The rate of eye preservation in survivors of recurrent RB was very low. Although 5-year OS in patients who underwent treatment for intraocular and orbital recurrence was high, it was low in those with metastasis. RB patients may need lifelong follow-up for recurrence and secondary malignancy. Treatment of RB patients requires a combination of modalities and should be individualized according to the patient’s disease.

## Data Availability

All data generated or analyzed during this study are included in this published article.

## Abbreviations

RB: Retinoblastoma
IVC: Intravenous chemotherapy
ASCT: Autologous stem cell transplantation
OS: Overall survival
NSE: Neuron-specific enolase
CNS: Central nervous system
